# Constrained to be (im)mobile? Refugees' and Asylum seekers' practices to integrate in restrictive socio-economic urban contexts in Northern Italy

**DOI:** 10.3389/fsoc.2023.1114394

**Published:** 2023-03-16

**Authors:** Iraklis Dimitriadis, Maurizio Ambrosini

**Affiliations:** ^1^Department of Sociology and Social Research, University of Milano-Bicocca, Milan, Italy; ^2^Department of Social and Political Sciences, University of Milano, Milan, Italy

**Keywords:** mobility, immobility, migrant agency, integration, COVID-19 pandemic, refugees, asylum seekers, Italy

## Abstract

This article comparatively examines forms of (im)mobility among refugees and asylum seekers (RAS) in coping with dispersal process, restrictive migration policies and local socio-economic characteristics in three cities of Northern Italy. Drawing on qualitative data, it sheds light on the everyday forms of (im)mobility of RAS to resist structural barriers limiting their opportunities to access jobs and welfare services. The Results show that people's capacity to overcome barriers depends upon individual characteristics and informal networks, and is shaped by particularities of local contexts. While people's regular legal status is considered an important resource in achieving goals, refugees and holders of international protection often have to adopt (im)mobility practices to access resources in contexts that do not facilitate their integration. This article highlights the inefficiency of integration and reception policies and advances the theoretical debate on the link between being (im)mobile and agency by calling authors to pay more attention to the (in)voluntary nature of spatial (im)mobility. Finally, it shows the ambivalent outcome of (im)mobilities in terms of agency, highlighting the implications for individuals before and during the COVID-19 pandemic.

## 1. Introduction

Labor market insertion and welfare services are two of the main domains determining refugee integration (Ager and Strang, 2008). The former concerns a range of services aimed to increase employability (e.g., vocational training, language courses, mentoring), so that people can achieve their goals and self-reliance. The latter includes the provision of basic needs (e.g., food, housing and clothing) and access to healthcare clinics, social service centers and rights to which all citizens are entitled. However, the implementation of integration policies is challenged by a series of structural, contextual and individual factors. These may include (a) policies regulating access to asylum and recognition of refugees' and asylum seekers' (RAS) qualifications and skills (Federico and Baglioni, 2021); (b) dispersal policies (van Liempt and Miellet, 2021); (c) local authorities' initiatives and bureaucracies' acts in favor of or against newly arrived people (Hinger et al., 2016); (d) the role of the civil society in facilitating or hindering integration processes (Dimitriadis et al., 2021); and (e) language barriers and psychological distress among newly arrived people due to the situation in their home country, experiences during the journey and waiting times for a decision on their asylum status (Federico and Baglioni, 2021).

This article examines how RAS cope with barriers to integration by exploring their daily practices of physical mobility and immobility.^1^ Drawing upon the perspectives of services providers, RAS themselves and non-participant observation at a migration service help desk and one reception facility, it emphasizes the factors shaping newly arrived people's agency and the implications of (im)mobility practices at the individual level before and during the COVID-19 pandemic. The focus on three cities that offer different opportunities in terms of jobs and services adds to the study of the factors shaping the agency of RAS by challenging views about the extent to which regularity in legal status implies successful integration trajectories. This article also contributes to the debate about the link between spatial mobility and agency, showing the importance of considering the voluntary or involuntary nature of mobility and immobility practices in examining (im)mobile people's agency.

The remainder of this article is organized as follows. The next section introduces the theoretical framework for this study and reviews previous works on (im)mobility practices of RAS, while the third section presents the methods and information about the cities where fieldwork research was conducted. The fourth section offers information about asylum policies and refugee integration in Italy. The fifth section presents the results of the analysis of the empirical material, while the final section provides some conclusive considerations and policy proposals.

## 2. The perspective of regimes of mobility and its application in studies on forced migrants

In analyzing the (im)mobility practices of RAS in Italy, this article adopted the framework of regimes of mobility introduced by Glick Schiller and Salazar (2013), according to whom the possibility of moving is embedded in unequal fields and relationships of political, social, cultural and economic power that unfold differently across the globe in various local contexts. Immobility (in the place of origin) is predominantly seen as involuntary due to constraints on people's opportunity and desire to be mobile (Schewel, 2020). In this respect, mobility and immobility are shaped by policies and (control) devices that may be applied differently on the basis of people's nationality, legal status and motivations for leaving their country of origin, for example (Faist, 2019; Sanò and Della Puppa, 2021). People's physical mobility and immobility should also be seen as a continuum in the sense that being mobile or staying put in a place can be interwoven.

The perspective of regimes of mobility highly criticizes assumptions about a linear link between physical and social mobility. Contrary to agentic dimensions attributed to “people on the move” by virtue of being mobile at the international level (Urry, 2007), and challenging overemphasis on national borders and states as points of departure and arrival, previous studies have shown that mobility across national borders can be seen as a consolation for those who cannot experience upward social mobility in the country of origin. Instead, staying put or remaining behind can indicate upward socio-economic mobility (Kalir, 2013). Similarly, other scholars have discussed how different (im)mobility patterns indicate the capacity of RAS to “shape and adapt daily routines and mundane social interactions to changing circumstances, precarious livelihoods” (Sigona, 2012, p. 51). These practices seem to generate “interstices”—that is, autonomous territorial, social and judicial spaces—that enable forced migrants to achieve some of their goals (Fontanari and Ambrosini, 2018). Remaining in the host society despite refusal of the asylum application should be considered an achievement, for example.

Departing from the above theoretical framework and concepts, a burgeoning literature has dealt with how forced migrants survive in the host society and overcome structural constraints by deploying practices of physical mobility or staying immobile. Refugees leveraging their legal status may engage in onward mobility relocating to another EU country due to better job opportunities, presence of relatives and ethnic ties, more generous welfare provisions, and to circumvent hostility and discrimination in the first destination country (Ahrens et al., 2016). Borri (2017) showed that those who obtain international protection in Italy can settle in Germany and may move back to Italy when they have to renew their documents. Mobility can be also internal for those who aim to access or maintain legal status by moving to localities across the national territory where public authorities are more favorable toward migrants (Dimitriadis, 2018) Contrarily, practices of physical immobility have been often associated to lack of agency in the sense that staying put in a place can be the result of vulnerability and social marginalization for those with irregular legal status or lack of knowledge of other territories (Sanò and Della Puppa, 2021). Yet, other research reveal contradistinctions in relation to the link between (im)mobility and migrant agency. Internal (temporary or seasonal) mobility may be due to the lack of employment in the place of residence, thus implying search for and access to informal jobs characterized by exploitative labor conditions (Cottino, 2021; Anderlini, 2022). Similarly, (periods of) immobility can indicate agency when people decide to stay in the same place to gain legal status (Wajsberg, 2020) or upward social mobility when refugees can maintain stable cross-seasonal work in the place of residence (Cottino, 2021).

Saying this, experiences of (im)mobility can vary among people, and patterns of mobility and immobility can overlap or intersect (Schapendonk, 2021). Migrant workers' (im)mobility (e.g., across places and jobs) are also embedded in diverse social and spatial relations; emotional ties with other family members or interactions with other people at the workplace can shape migrants' decision-making in terms of mobility (Zampoukos, 2018; Dimitriadis, 2023). In addition, external shocks, such as the COVID-19 pandemic for instance, can even constrain people's mobility or open new opportunities (Sanò and Della Puppa, 2021; Anderlini, 2022).

Overall, previous research put little emphasis on the factors that determine people's ability to get by or improve their lives, with the exception of legal status, which is considered a key element in shaping migrant agency. Our research fills this gap by reflecting on such factors. In addition, it elaborates on the (in)voluntariness in migrants' actions. Previous studies do not take into consideration the distinction between the voluntary and involuntary character of immobility (Carling, 2002; Schewel, 2020) when accounting for their outcomes in terms of agency. People's “stasis” can be due to constraints on movement or can reveal a desire to stay or can be something in between, for instance. In the same vein, Ottonelli and Torresi (2013) highlight the importance of analyzing migrants' acts under the lens of voluntariness, as this notion enables moving beyond normative theory of migration seeing people as either victims or villains (Anderson, 2008). In reflecting, therefore, on the voluntary or involuntary nature of the spatial immobility of RAS, this article complicates the debate about the link between being mobile and agency.

## 3. Methods and contexts of inquiry

This article is based on empirical material that collected through 50 interviews with service providers including managers, social workers and volunteers in formal and informal reception facilities; employees of (private and public) employment centers; representatives of third sector organizations (TSOs) promoting vocational training projects; trade unionists; lawyers with expertise on immigration; and refugees and asylum seekers (Table 1). The important role civil society has played in facilitating the settlement and integration of migrants in recent years (Dimitriadis et al., 2021; Ambrosini, 2022) led us to opt to explore the integration of RAS from the perspectives of service providers. However, these data were triangulated through eight interviews and informal discussions with RAS and ethnographic material collected through instances of non-participant observation at a migration service help desk and an informal reception facility in Como. The sampling of service providers was purposive. We initially contacted representatives of pro-migrant organizations known to be active in providing service to RAS. These people, then, introduced us to other colleagues or people engaged with migrants. The selection of research participants was based on the heterogeneity of types of CSAs and their services toward migrants. RAS were contacted thanks to the intermediation of service providers.

**Table 1:** The sample.

**Participants**	**Busto Arsizio**	**Como**	**Milan**
Reception center managers	3	2	2
Social workers	3	5	7
TSO and independent volunteers	5	2	3
Trade unionists	1	1	2
Religious actors	1	1	1
Lawyers	1	1	1
Refugees/Asylum seekers	3	4	1
Total	17	16	17

Interviews were conducted in the Italian language from May 2019 to May 2021, and typically lasted between 40 min and 2 h. Those conducted before March 2020 took place face to face in public spaces or at the venues of the associations, whereas most of those done after the pandemic outbreak were carried out *via* video communications platforms or telephone. The difficulty of accessing RAS during the pandemic influenced our initial sampling strategy, thus leading us to mainly focus on service providers' perspectives and adopt alternative techniques of collecting data. More precisely, sporadic instances of non-participant observation have been possible in periods in which measures against COVID-19 transmission were limited or lifted. All participants were informed about the scope of the research and gave their consent to participation, audio registration and processing of personal data. The process of data collection and analysis was approved by the ethics committee of our university. Answers were anonymised, coded and analyzed using QDA Miner, which facilitates thematic analysis of qualitative data. After familiarizing with data and identifying items of interests, we generated codes capturing both the semantic meanings and latent assumptions underpinning the surface meanings (Braun and Clarke, 2006). Then, we organized codes into themes and proceeded with the analysis of data extracts. For this article, we used the most compelling examples related to our research questions and literature on (im)mobility practices.

Data were collected through fieldwork carried out in three cities: Busto Arsizio, Como and Milan. All of these cities are located in the region of Lombardy in Northern Italy, where the majority of migrants are concentrated (almost 22% of migrant population living in Italy reside in Lombardy: www.istat.it). In addition, 45.6% of asylum seekers and people under humanitarian protection reside in Northern Italy (www.regioni.it). The selection criteria for these specific cities included: (a) the stance of local authorities and communities toward RAS, (b) labor market opportunities and (c) geographical position. Therefore, the selection enabled us to explore the notion of mobility and immobility in two mid-sized satellite cities close to a metropole like Milan.

Milan (1,406,242 residents) has been selected as a big city ruled by a local authority with a positive stance toward refugees (Artero and Fontanari, 2021). Twelve municipalities across its province hosted almost 800 refugees at SAI facilities in 2021 (www.retesai.it). As one of the richest urban centers, migrants can easily access (informal) jobs in the service and construction sectors. The stance of the municipality of Busto Arsizio (83,679 residents) toward RAS can be characterized as indifferent or hostile; local authorities have never adhered to the SAI network. In addition, the arrival and settlement of RAS at a CAS facility in the city generated conflicts and mobilization among citizens. Four municipalities close to Busto Arsizio (in the Province of Varese) run SAI facilities that hosted 99 people in 2021, whereas 45 asylum seekers are hosted in CAS centers located in the city of Busto Arsizio. Most job opportunities for migrants are available in the service and industrial sectors of the economy. The selection of Como (83,679 residents) was based on its particularity as a border city, as it serves as locality for short-term stay for those who aim to move to other European countries by crossing the northern Italian borders. Its municipality had a hostile or intolerant stance toward RAS (Dimitriadis and Ambrosini, 2022). No SAI facilities were available in this province until 2021, whereas eight CAS located in Como hosted around 330 asylum seekers in 2021. Migrants can access employment in the service sector, while tourism offers other job opportunities.

## 4. Reception and integration of RAS in times of restrictive asylum policies in Italy

In dealing with the increasing number of people arriving to seek international protection since 2015 and facing municipalities' reluctance to be involved in the reception of RAS, the Italian government introduced a complementary reception mechanism. In addition to the ordinary protection system for asylum applicants and refugees run on a voluntary basis by municipalities with the possible engagement of third sector organizations (SPRAR network, then named SIPROIMI and now SAI), the law n. 142/2015 provided the opening of emergency reception centers (CAS) for those who could not be hosted within SPRAR facilities upon arrival. These new structures are managed by various private actors, such as non-governmental organizations (NGOs), hotel owners and other conventional employers, without the engagement of municipalities. However, due to the increasing number of newly arrived people and the scarce willingness of many local governments to join the SPRAR network, CAS became the main reception facilities hosting between 75 to 80 per cent of asylum seekers in Italy in the period 2014–2018 (Campo et al., 2020). These reception faculties were thus called to align the quality of services for the basic needs of RAS and their integration to those offered by the SPRAR system (Dotsey, 2022).

Integration of (forced) migrants in Italy can be characterized as decentralized, as the national government defines the minimum standards and key priorities of people's insertion in the society, whereas regional and local institutions promote and implement a series of measures and policies. Services for forced migrants' integration are often provided through third-sector organizations (Scholten et al., 2017). In this context, the goal is to promote inclusion and integration through access to the labor market, basic rights and welfare services (e.g., housing, healthcare), education, language courses and civic participation.

The pathways of the integration of RAS were challenged due to the implementation of new restrictive asylum policies. The law no. 132/2018 (the so-called Salvini or Security decree) excluded asylum seekers from ordinary reception facilities, thus making CAS the only structures that could host them until the final judgement upon their application was made. In other words, only people with a legal status could access the SAI network where they could then stay for 6 months. The same amendment also provided reductions in fares that cover asylum seekers' needs, namely from 35 reduced to 20 euros per day, which additionally limited integration services previously offered in CAS centers, such as Italian language courses, orientation to the labor market and psychological and medical assistance. Being deprived of integration services while awaiting the decision upon asylum requests, self-reliance among refugees and holders of the international protection status could therefore hardly be achieved through a 6-month project offered by the SAI network; people's independence was expected to be lower in localities where the labor supply is limited. Moreover, with the new amendment, the possibility to access and maintain the status of humanitarian protection was narrowed only to those facing serious health problems, coming from countries suffering natural disasters or those who had been abused. People who are not able to prove that had suffered persecution and came from unstable or non-democratic countries in which they are equally at risk are now excluded. Therefore, the number of refused asylum seekers has increased over the years^2^ (Dimitriadis and Ambrosini, 2022), including both newly arrived people who cannot access international protection and holders of international protection who failed to renew their status or convert it into a stay permit for work reasons.

The Italian dispersal policy seems to further constrain the integration of RAS. The allocation of asylum seekers across the national territory has happened in a quasi-random basis (Campo et al., 2020), whereas the majority of SAI facilities are concentrated in southern Italy, where job opportunities are lower relative to northern Italy. Settlement into socially deprived urban and rural areas can also affect people's integration, as RAS are more likely to experience hostility and institutionalized marginality (Sanò and Della Puppa, 2021; van Liempt and Miellet, 2021). Similarly, isolated (mountainous) areas do not seem to favor the integration of RAS (Cottino, 2021), given the limited (seasonal) job opportunities and lack of services targeting migrants in these areas.

In light of this information, we now proceed with our findings. The next section is divided into two sub-sections: the first concerns (im)mobility practices related to work, while the second focuses on migrants' efforts to access welfare services.

## 5. Findings: Mobility and immobility practices of RAS to access employment and welfare services

### 5.1. Being (im)mobile to access jobs

#### 5.1.1. Daily commuting from small cities to Milan to access (precarious) jobs

According to our informants, a common mobility practice that emerged from our fieldwork concerns daily commuting from Busto Arsizio and Como to Milan for work reasons. While those living in Milan can easily access precarious or (informal) low-wage jobs (mainly as couriers for food delivery companies or other odd jobs), people residing in small cities move to face lack of employment opportunities in their place of residence:

When an asylum seeker becomes a worker who receives a higher salary than the annual social allowance, he cannot remain in the reception center. So, some of those getting these occasional jobs leave the reception center because they have exceeded the threshold. But, then, they may lose their jobs and want to re-enter the reception again. (Trade Unionist, Busto Arsizio)

You cannot see refugees and asylum seekers anymore in the city. You can only see them go out and enter their homes. They work as riders with those big square-shaped backpacks; they get the train, go to Milan and work. (Volunteer, Busto Arsizio)

Our researcher (person collaborating with the association) notes that the probability of finding a job – maybe at the end of the vocational training period – is too low, and this is known among asylum seekers. (Volunteer, Milan)

As long as asylum seekers are waiting for a decision about their application (Ramachandran and Vathi, 2022), they may opt to be mobile to earn more money through precarious employment than the funds coming from the monthly pocket money (€200–250). Acceptance of such jobs allows RAS to cover their actual (survival and sending money back to their families) or future needs (e.g., plans to move onward). This may also be due to perceived low probabilities of getting a (good) job through vocational training in the long term. This is also common among people who have already received legal status and did not find stable employment once their time within the institutional reception facilities was over. However, everyday mobility to Milan is not without implications. High levels of flexibility and insecurity characterizing platform economy (Tassinari and Maccarrone, 2020) and other odd jobs can challenge people's trajectories (e.g., unemployment and inability to afford housing), as many of them lose the right to reception. In addition, using public means of transportation to reach Milan not only entails economic costs, but also inconvenience and risks when workers have to return back home late due to the limited capacity for bicycles on train cars. This means that riders who cannot find a place on train cars have to stay overnight in Milan or return home by bike, thus having to travel long distances. This implicates exposure to risks when using provincial roads without lights after a long, tiring day at work. Rules to stop the spread of COVID-19 on public transport increased this problem, whereas those who work as undeclared workers risked receiving fines upon police control during lockdown periods. In addition, platform workers were particularly exposed to COVID-19, without paid sick leave or sickness benefits (ILO, 2021).

Abandonment of integration projects and insertion into precarious jobs in Milan through daily commuting seem to be linked to the outbreak of the current pandemic, too. The following narrative is telling:

Most of the companies we collaborate with are in the tourism sector, restaurants and hotels and so on. The Region (of Lombardy) stopped activating new internships during the pandemic. Some projects were available but at distance… but remote internships for our guests are very few, in the sense that most (of the jobs people got trained in) are also very physical, manual or, in any case, jobs that require (trainees') presence. […] Compared to other years, there have certainly been 70–80% fewer job entries due to the pandemic (in 2020). (Social worker, Como)

COVID-19 can, paradoxically, be seen as a catalyst for daily mobility for work. The effects of the pandemic on the hospitality sector were of great magnitude, thus impacting the integration trajectories of RAS. Limited efficiency in offering remote training constrained RAS to abandon vocational training projects and access precarious jobs in big cities like Milan. This result was very common in places such as Como, which are based on tourism sector. However, not all asylum seekers are expected to react in the same way. Instead, many of them stayed put in their place of residence:

Unfortunately, many of these guys spend all their day closed up at home doing anything. (Volunteer, Como)

I don't want to do this job (rider). All those I consider as work experience are in logistics; and I want to do this (kind of job). […] During the lockdown I got a driving license. My association helped me to make the registration, but I myself paid around 1,200 euro to get it. (Asylum seeker, Busto Arsizio)

Everything is now stuck for women (due to COVID). We achieve few job insertions, always in the cleaning sector, or less in care services. Some of them are interested in aesthetics. But this kind of vocational training requires quite a bit of time and is expensive, and therefore it is not easily accessible to them. (Social worker, Como-Milan)

These quotations suggest that immobility can have different outcomes according to one's gender or capacity to mobilize resources. Lockdown periods meant a period of stagnation for those who were not be able to access vocational training programmes or jobs. Difficulties in holding remote training, coupled with the gendered nature of local labor markets, left little space for women to access job opportunities. However, others could use their time to increase their employability, regardless of their legal status. The participant in the above example was a refused asylum seeker who repeated his application. Therefore, people's ambitions and capacity to access resources (financial capital) (Simşek, 2020) can give spatial immobility a different meaning.

Overall, among refused asylum seekers, staying put in Italy can be seen as a form of resistance to deportation policies. As our research participants argued and previous research has shown, refused asylum seekers often remain in the Italian territory by relying on social ties with national fellows and civil society to get by (Dimitriadis and Ambrosini, 2022). When thinking of the possibility of regularization through amnesties (Bonizzoni and Hajer, 2022), this kind of immobility can even indicate improvement of one's life.

Looking now beyond the effects that Italian restrictive policies and the pandemic had on the integration of RAS, discrimination is another structural element that triggers everyday mobility among RAS from small cities to Milan:

In Busto Arsizio, for example, you don't see any black waiters—that is: we are not in Milan or London. Here, they (black people) can only work as dishwashers or kitchen helpers in restaurants and bars; they don't stay in the room where clients eat. Going around the city and having a look is enough to see this. We have a small-town mentality from this point of view. (Social worker, Busto Arsizio)

This excerpt suggests that small cities can induce movement to multicultural cities due to discrimination in the economic sectors in which migrants can find employment opportunities. With reference to the pre-COVID-19 period, almost all service providers pointed out that restaurants and cafés did offer job opportunities. However, when cities are not open to diversity (Pastore and Ponzo, 2016) migrants can be excluded from jobs that entail their being visible to and in contact with clients, thus indicating a mismatch between job opportunities and political attitudes by the local population. This kind of mobility, hence, can be seen as enforced due to hostility in local contexts that makes these places not “inhabitable” (Sanò and Della Puppa, 2021). In the following section, we show that spatial mobility can also include periodic or occasional movements across the Italian territory and beyond.

#### 5.1.2. Seasonal or occasional mobility to access agriculture jobs and transnational movements

RAS often move to Southern Italy to take on employment in the agriculture sector (Cottino, 2021; Sanò and Della Puppa, 2021). This mobility pattern is confirmed in our case studies, too, but it may concern localities across the whole Italian territory.

Many homeless people who lose their status leave Como to find employment in other cities. Two guys went to work in Verona for a couple of weeks. Another guy went to Caserta (Southern Italy) to find a job through a cousin of his. However, they often turn back to Como because they realize that moving to another place is not that easy if you do not know anybody. Here, they can at least find a roof under which to stay and food. (Volunteer, Como)

Regardless of their legal status, RAS may leave their place of residence during the spring months and return when the crop of agriculture products ends. By relying on co-ethnic brokers (Ambrosini, 2017), seasonal or temporary mobility practices enable people to survive or fund their plans, despite the implications of the precarious or exploitative nature of work in agriculture. People tend to return back to the place where they have social ties and can access welfare services though civil society's action, as analyzed below. Therefore, such mobility practices do not seem to improve one's conditions over the long term.

Despite rigidity in border controls across Europe since 2016, RAS can also be mobile across national borders to find employment opportunities (Della Puppa et al., 2021). Asking a volunteer in Como to put me in contact with a refugee who resides in Como, he told me:

He is not in Como this period. He often goes to France, where there are some friends of his. He is not stable in the city, but he usually returns here. He follows some job opportunities and his life is like this. (Volunteer, Como)

In this case, transnational migrants can undertake work trips for different periods of time to access job opportunities in third countries and explore the possibility of settling there (Dimitriadis, 2023). This practice allows RAS to get by and possibly to create the conditions to settle in a new place. Transnational mobility can be also undertaken by (refused) asylum seekers, but with some implications, as the following ethnographic notes reveal:

I'm assisting a discussion between a lawyer volunteering at a pro-migrant association in Como and a refused asylum seeker who intends to submit a repeated asylum request. Lawyer: “The problem is that you've been away from Italy in the last months and this doesn't help you. If you cannot demonstrate that you're in Italy, your application has no chance of being accepted. Why did you leave Italy?”

Refused asylum seeker: “I went to (name of European country) just for work. I have a cousin there and I went to earn some money.” *(Ethnographic notes at the migration service help desk, 9 April 2021)*

As shown in previous research (Wajsberg, 2020), the importance of staying put in one place while waiting to receive legal status was recognized by well-informed migrants, who were able to receive information about the factors determining the success of their application. In other words, mobility can contribute to the reduction of one's chance of holding legal status, thus indicating the negative side of transnationalism that has usually been connected to agentic dimensions (Dimitriadis et al., 2021). Immobility can also be a solution leading to continuity in employment, as the following section reveals.

#### 5.1.3. Spatial immobility to face restrictive asylum policies

While staying put in one place among RAS has been often linked to the inability to acquire or maintain a stable legal position, the following excerpt tells a different story:

One woman who obtained international protection moved from Como to a SPRAR in the province of Varese, and she could not keep the job in the hotel where she worked in Como. This is a problem. […] Other people reject moving to SPRAR facilities and remain here (in Como), organizing their pathway on their own, avoiding moving to other places. (Social worker, Como)

Vocational training courses were not at all designed for female asylum seekers. There were training courses for electricians, for mechanics, for purely male jobs. Furthermore, the other difficulty is that they (women) often have to take care of the children, and they do not even manage to have the time, the opportunity to go to school to learn Italian… what I'm trying to do is to incentivise them to develop forms of solidarity. Now, there are some language courses, and one mum looks after another mum's children, so they can participate at the courses. Some of them are illiterate, so it's even more difficult to participate in language courses. […] the other problem is that they're waiting to access the SPRAR system: one mum and her daughter have been waiting for two years to access the SPRAR network, but unfortunately there are only few SPRAR centers for families in the Monza–Brianza zone. (Social worker, Como and Milan)

In the first case, immobility can be seen as a form of resistance to dysfunctionalities in the formal reception system among refugees and holders of international protection. People may reject uprooting themselves from the place where they have already created social ties and found stable employment. New movement may be considered risky in terms of integration (e.g., labor market, housing, ties with locals). This pattern was common in the municipalities of Como and Busto Arsizio, which have never adhered to the SAI network. In addition, the lack of policies reflecting women's (and parents') needs conditions people's access to the labor market. Mobility to access SAI centers in different localities is seen as even more counterproductive for women's integration, in the face of the major difficulties they confront when they take care of their children. In this context, immobility indicates a practice to get by, whereas forms of solidarity developed among women (and incentivised by civil society) can contribute to coping better with structural barriers. Having accounted for (im)mobility among RAS to access or secure employment, we now move to the next section dealing with people's practices for accessing welfare services.

### 5.2. (Im)mobility practices to cope with lack of welfare services

#### 5.2.1. Mobility to access affordable (informal) housing

Spatial mobility is also adopted by RAS who cope with difficulties to access housing. A common practice for finding a housing solution is to rely on networks of friends and acquaintances:

When men leave the reception facilities, they organize themselves to rent apartments together; I'm talking about guests of the same reception center who leave and share the rent with other people. Thanks to the network of compatriots, friendship networks, many guys leave in this way. (Social worker, Busto Arsizio)

This kind of mobility not only concerns asylum seekers who cannot access formal reception facilities, but also refugees or holders of international protection status who often cannot enjoy economic independence after the reception period at CAS or SAI facilities. Despite the importance of ties with fellow nationals in accessing housing (Ambrosini, 2017), RAS may face further hardships in accessing the private housing market:

I heard some bad (offensive/discriminatory) things... (there's) discrimination while seeking a house. I asked many friends of mine. They ask around and they (owners) reply: “no, no I don't rent out the house to Africans”. […] at the end, always Valerio and other friends managed to find this house (where he actually lives) for me. The house owner said me that he has known Valerio for 10 years, and this is why (I got the house). (Refugee, Busto Arsizio)

This quotation underlines the importance of the existence of social ties with locals in the search for housing after the period of institutional reception. In the above case, the key person was a social operator working in vocational training programmes who tutored the refugee in the search for employment. In other words, entering the networks of local native people allows refugees to withstand racial discrimination as locals can stand as guarantor for the character of people to whom house owners rent out their property (Ravn et al., 2020). Beyond racism or discrimination, though, refugees may be rejected access to housing due to the precarious or informal character of their employment, which gives no guaranty to house owners.

Spatial mobility can become recurrent when housing solutions share a temporary or informal nature among people with few resources:

In the city of Varese there is perhaps one dormitory, but the places are insufficient to respond to the needs of the homeless population. Instead, Milan has a much higher number of available places. In Milan, there are also food and soup kitchens, shower services—there are all the services for marginalized people; everything is much more structured; and, therefore, we give needy people addresses in Milan (where homeless people should go and which persons have to contact). (Social worker, Busto Arsizio)

Homeless migrants may move to cities where housing services are available to all people regardless of their legal status. Many of those who exit the institutional reception system or those who lapse into irregularity may opt to move to Milan to access temporary solutions at minimum standards in a system of services that provides forms of “poor relief” (Leerkes, 2016). This is because the city where they live and the Province of Varese, in general, do not dedicate adequate resources for the protection of homeless people. This kind of mobility is facilitated by information circulated through TSO workers. One research participant who lost the right to international protection and whose appeal to this decision is still pending, confirms the allegations of the social worker in the previous quotation:

Interviewer: why did you decide to come to Milan?
Respondent: it's not that I decided to do so. The first time I was in a CAS in [name of a satellite city close to Milan]. Then, when I entered the SIPROIMI system, I had to move to [name of another satellite city close to Milan]. When I lost the international protection, [an Italian friend of his—a social worker] gave me the contact of a woman working in the welfare facilities for homeless people in Milan. That's why I live here now. It's not my choice. (Asylum seeker, Milan)

The mobility of homeless people is therefore linked to survival rather than being a planned and desirable action. Due to municipalities' indifference or inability to contribute to the reception of RAS, enforced mobility between different places across Italy may affect the integration of RAS because it entails the uprooting of people who are not able to maintain the social ties that they have developed in local communities (Ager and Strang, 2008; Simşek, 2020). While lack of forms of “poor relief” should also be expected in the case of Como, recent research tells a different story (Bonizzoni and Dimitriadis, 2021). Due to the geographical position of this city, civil society actors have been particularly active in providing accommodation to homeless migrants since 2016. Dealing with thousands of people who traveled to, remained entrapped in or turned to (the so-called “Dubliners”) this border city, civil society organizations have undertaken innovative practices to offer housing and integration prospects to people without financial resources and with unstable lives. Available structures offer accommodation on a permanent or temporary basis, thus allowing migrants to stay put and work in the city or engage in circular mobility, as argued above. This is not the case in Busto Arsizio. Social workers in this city often claimed that they do not see anymore those who lapsed into irregularity.

As already argued, the distance between one's dwelling and workplace implies daily commuting. In other cases, people who exit reception facilities often have to leave the city in which they used to live to afford accommodation. Although getting public means of transportation to the workplace on a daily basis may be a solution, this is not always feasible:

Some guys have jobs outside Como, in hotels, and they have to remain to sleep there. At that point, they have to make a choice, in the sense that the Prefecture, unfortunately, does not allow people to sleep outside the reception facilities. So, some guys made the choice to reject job opportunities or exit the reception system because it was not possible to reach hotels. […] We're advising those who have an internship contract to save money to invest it for those expenses (e.g., getting a driving license) that we cannot fund. (Social worker in receptions facilities, Como)

This quotation suggests that access to employment can be constrained due to the distance between reception facilities and the workplace, which generates dilemmas among RAS about missing jobs opportunities or the right to accommodation (and, generally, reception) in case they cannot maintain employment positions. Seasonality in jobs also implies that RAS have to find housing solutions for the months they remain unemployed. Some RAS can overcome such barriers, yet:

Respondent: I own a car, I bought the car 2 years ago so I went to work by car when I did the training at [name of company]. The companies are located far from the city, they are in the industrial area. Every time I sent out a resume, they asked me for my driving license and car, so I sacrificed myself and got my driving license and car.
Interviewer: It's not easy to get the driving license, were you able to drive?
Respondent: I knew how to drive, I drove for many years (in his place of origin), but the theoretical part (of the driving exam) was not easy, but I learned a bit quickly anyway, because I am quite good at studying. (Asylum seeker, Busto Arsizio)

This quotation highlights that financial (Simşek, 2020) and cultural resources (good language and driving skills) are very important in coping with constraints imposed by dispersal policies and opportunities in local labor markets. Comparing the three case studies, although RAS in Milan face fewer problems with daily commuting while residing at reception facilities (thanks to the efficiency of public transportation and the availability of jobs around the city), the situation changes when they lose the right of accommodation or opt to abandon state reception facilities. This is because RAS have to move to satellite cities due to the high cost of housing in Milan. Next to (im)mobility practices to deal with housing issues, RAS can opt to be (im)mobile to access better treatment in relation to bureaucratic procedures, as the next section shows.

#### 5.2.2. Mobility to migrant-friendly cities to access administrative services

Mobility practices are also adopted when RAS have to cope with hardships related to bureaucratic procedures. This can concern the internal mobility of people who already reside in Italy (Sanò and Della Puppa, 2021) or those who have just arrived after having crossed the Italian territory borders:

They are mainly Pakistani and come from Udine, Trieste and someone from Gorizia. It works by word of mouth. In fact, I found out that they (Pakistani asylum seekers) are advised to come to Como (through information they get) on a Pakistani website. They arrive here because one thing we do is to help them make the first asylum request, and at the same time we look for a reception center. […] Having lawyers (who help newcomers), the insertion in CAS is faster. Or we try to find a housing solution. […] in addition, the police headquarters collaborate with us, so this helps. […] In Gorizia or in Trieste you have to wait for 1 month before you have an appointment at the police headquarters; here, it takes just 2 weeks. (Social worker, Como)

Benefiting from the gap between national policies and actual implementation, settled or newly arrived people can access information that provides them with better chances to regularize and/or access services and rights (Van der Leun, 2003; Dimitriadis, 2018). This is not only the case in Como, as our participants located in Milan confirm a similar trend in relation to specific police headquarters in Milan. Mobility to enjoy more favorable treatment by local bureaucrats and services offered by civil society actors is even more beneficial when combined with the possibility of insertion into the labor market:

Most of Pakistani people come to Como because they know that they will find job in the fruit market, loading boxes or selling roses. However, they have limited chances to get regularized. (Social worker, Como)

Relying on a well-established Pakistani community in Como (Bonizzoni and Marzorati, 2015) or other ethnic communities in Milan, newly arrived people move to these cities not only to access services, but also to insert themselves into niches of the (informal) local labor markets which may be beneficial for their integration in the long term (Bonizzoni and Dimitriadis, 2021). Another practice of (im)mobility we identified relates to access to health services and is presented in the final section.

#### 5.2.3. Forced mobility to receive health services in Milan

The cutting of funds dedicated to services for people suffering psychological or psychiatric disorders has led people to move to Milan to receive medical care:

What is lacking in the territory (Province of Varese) is the accompaniment of people facing psychological and psychiatric vulnerabilities. Especially in the CAS, we are in difficulty when we find such cases. […] As for the SIPROIMI, we collaborate with the private sector. This is quite important, but not all SIPROIMI projects have a sufficient budget to provide such services. (Social worker, Busto Arsizio)

We rely on the psychiatric department of [name of hospital] in Milan. There are some professionals in our territory, but they are few. It is certainly a limitation (for people's integration) in this area. (Social worker, Como)

Faced with the lack of healthcare services, RAS located in Busto Arsizio and Como often have to move to Milan to receive medical care. This last quotation confirms previous research on the role of medical centers and NGOs based in Milan that provide services to (irregular) migrants by furnishing non-urgent medical care, including psychological and psychiatric services (Ambrosini, 2017). Inclusive policies and the active engagement of civil society in asylum governance seem to remedy the shortcomings of state policies in the health sector.

## 6. Conclusion

This article examined the forms of spatial mobility among RAS in coping with structural constraints on their integration paths in three Northern Italian cities. It mapped the different ways in which RAS deploy mobility and the situations in which they stay put, highlighting the factors shaping these acts, as well as the outcomes and implications at the individual level. In doing so, it contributes to the analysis of the agency of RAS, and the theoretical debate on the link between spatial (im)mobility and their agency by calling attention to the (in)voluntary nature of being (im)mobile.

This article advances knowledge about individual and contextual factors that enable RAS to navigate the reception system better. Financial capital (e.g., the ability to get a driving license or buy a car), knowledge of the local language or previous skills (e.g., ability to drive) and social ties that can provide useful information (e.g., about the regularization process, jobs and housing) enhance people's capacity to cope with structural constraints. While legal status is considered an important factor in favoring integration processes, this study reveals that holders of international protection and refugees often (have to) undertake similar (im)mobility practices as (refused) asylum seekers. This indicates the failure of the integration and dispersal policies of the Italian reception system and is linked to the specificities of the localities where migrants reside. Some of the contextual factors that hinder the integration of RAS are considered to be discrimination on a racial basis and low demand in the labor market. Lack of efficient transportation services in small cities on the one hand and the high cost of living in big cities on the other can also obstruct people's insertion in the local labor market. The limited welfare services offered by municipalities and the distance between place of residence and workplace are two factors that limit people's agency, regardless of the socio-economic characteristics of the local contexts. Instead, high labor demand and the presence of a proactive civil society in favor of RAS facilitates people's settlement and integration. The geographic position of a city can either enable or hinder people's integration.

In light of these factors and considering the effects of COVID-19 pandemics on people's lives, it can be argued that this external shock triggered different practices of (im)mobility that had heterogeneous results in terms of agency. Although most integration programmes were suspended, some RAS opted to be mobile to access income through odd jobs, others increased their employability (e.g., language courses or vocational training), whereas other people remained unemployed and without available alternatives waiting for the ends of COVID-19 restrictions.

Looking now at the different types of mobility and immobility among RAS, this article elaborates on the discussion about the connection between (im)mobility and agency. While previous studies that adopted the “mobility regimes” approach challenged the linear relation between immobility and downward social mobility (Kalir, 2013; Wajsberg, 2020; Sanò and Della Puppa, 2021), this article advances the discussion on the meaning of (im)mobility in terms of agency by calling attention to the voluntary or involuntary nature of (im)mobility practices. On the one hand, the mobility practices of RAS in the host society often seem to be enforced by structural dynamics such as restrictive migration policies. Talking about spatial mobility for work reasons or to access welfare services, this might be considered involuntary, as it often entails poor employment conditions or reflects the lack of jobs, vocational training or inadequate integration projects in places where migrants live. On the other hand, staying put in the host society, even among those who hold an irregular legal status, can be considered voluntary or chosen immobility, as part of their mobility trajectories that goes against border policies. In other words, immobility can be seen as a strategy of resistance against the reinforcement of external and internal borders (e.g., local policies of exclusion) and can be considered as the other side of their actual plans of being mobile. Similarly, refugees can opt to stay put in a place to cope with the lack of integration projects where they live.

All in all, even under the constrained situations in which the RAS may find themselves in, being (im)mobile and accepting poor and harsh work conditions cannot be conclusive evidence of their being compelled to do so (Ottonelli and Torresi, 2013). Rather, it may be seen in the light of one's access to resources and ability to mobilize them in order to get by or improve her/his life. Therefore, instead of merely criticizing the linear connection between voluntary mobility and agency, future research should consider people's (im)mobility preferences to provide a better understanding of the interplay between being (im)mobile and being able to resist or overcome structural barriers.

## Data availability statement

The raw data supporting the conclusions of this article will be made available by the authors, without undue reservation.

## Author contributions

ID contributed to the writing of all sections of this article. MA contributed to the writing of the conclusions, reviewed the whole article, accessed funding for the realization of this research, and supervised the collection of the empirical material on which this article is based. Both authors worked together for the conception and organization of this article.

## Footnotes

^1^Following Schewel (2020: 329) definition, we see immobility as “spatial continuity in an individual's center of gravity over a period of time”. This is to say that immobility is related to both spatial and temporal frames (e.g., residential or throughout the life course). Immobility cannot be absolute in the sense that all people are engaged in everyday forms of movement.

^2^This trend has slightly changed as a result of the implementation of Law 173/2020 that reintroduced a special protection permit concerning people within the following categories: calamity, elective residence, acquisition of citizenship or stateless person status, sporting activity, artistic work, religious reasons, assistance to minor.
